# A rectal ulcer caused by hydrogel spacer insertion: A case report and review of the literature

**DOI:** 10.1002/deo2.70036

**Published:** 2024-12-02

**Authors:** Sen Yagi, Moyu Kawano, Keitaro Kawasaki, Takatoshi Murakami, Jiro Miyaike, Shinya Furukawa

**Affiliations:** ^1^ Department of Internal Medicine Saiseikai Imabari Hospital Ehime Japan; ^2^ Health Services Center Ehime University Ehime Japan

**Keywords:** hematochezia, hydrogel spacer, prostate cancer, radiotherapy, rectal ulcer

## Abstract

A 75‐year‐old man presented with hematochezia. He had been diagnosed with prostate cancer (stage 1) 1 month previously and had undergone gold marker injection and hydrogel spacer insertion 3 weeks previously to prepare for radiotherapy. Hydrogel spacer insertion is a safe procedure that can prevent the side effects of radiotherapy for prostate cancer. A computed tomography evaluation identified a low‐density area that extended from the prostate to the rectal wall. Magnetic resonance imaging of the abdomen revealed the hydrogel spacer between the anterior rectal wall and prostate. A colonoscopy revealed an approximately 2 cm ulcer in the rectum. The patient was diagnosed with a rectal ulcer with bleeding caused by hydrogel spacer insertion. Conservative follow‐up was performed, and his condition improved over time. Radiotherapy for prostate cancer was initiated 4 months after hydrogel spacer insertion. The patient has not experienced any abdominal symptoms such as bloody stools since that time. Because the incidence of prostate cancer is increasing, the number of such cases is likely to increase in the future.

## INTRODUCTION

Radiotherapy (RT) is a standard treatment for prostate cancer, which is frequently encountered worldwide. During RT, high doses of radiation are administered to the rectum and bladder, which surround the prostate; this can result in side effects such as radiation proctitis and radiation cystitis.^1^ However, hydrogel spacer insertion can be performed to prevent these side effects. We describe a case of a rectal ulcer that developed after hydrogel spacer insertion. Such cases are expected to become more common with the increasing incidence of prostate cancer. Additionally, we reviewed the literature regarding rectal ulcers attributable to hydrogel spacer insertion.

### CASE REPORT

**FIGURE 1:**
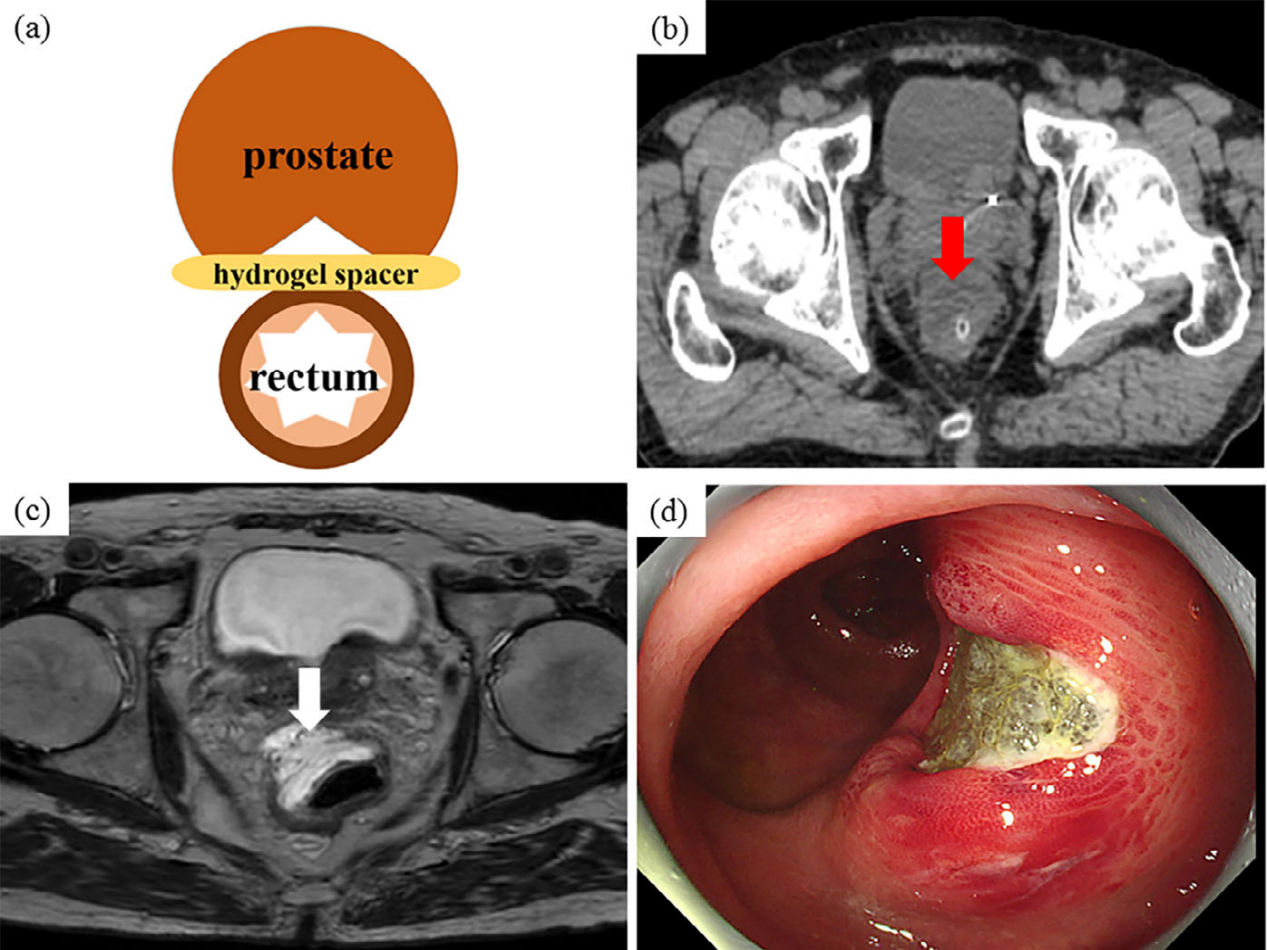
The hydrogel spacer is inserted transperineally between the anterior rectal wall and prostate fascia (a). Computed tomography image (b) of the abdomen shows a low‐density area that extends from the prostate to the rectal wall (red arrow). Axial T2‐weighted (c) and sagittal T2‐weighted magnetic resonance imaging images of the abdomen show the hydrogel spacer in the area between the anterior rectal wall and prostate as well as within the rectal wall (white arrow). An image obtained during colonoscopy (d) shows an approximately 2 cm ulcer in the rectum with bleeding around the hydrogel spacer.

**FIGURE 2:**
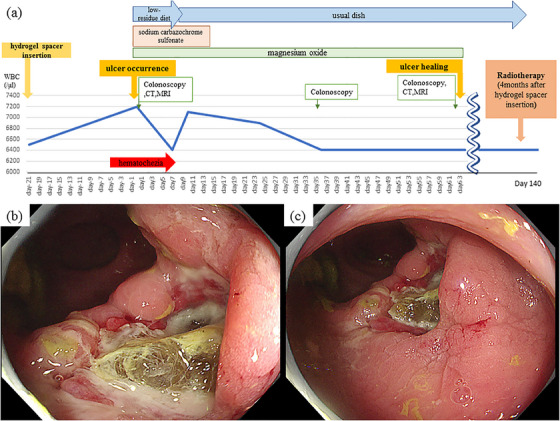
Clinical course of the rectal ulcer caused by hydrogel spacer insertion (a). Images obtained during colonoscopy 1 month after the diagnosis. The ulcer shows signs of improvement (b, c).

**FIGURE 3:**
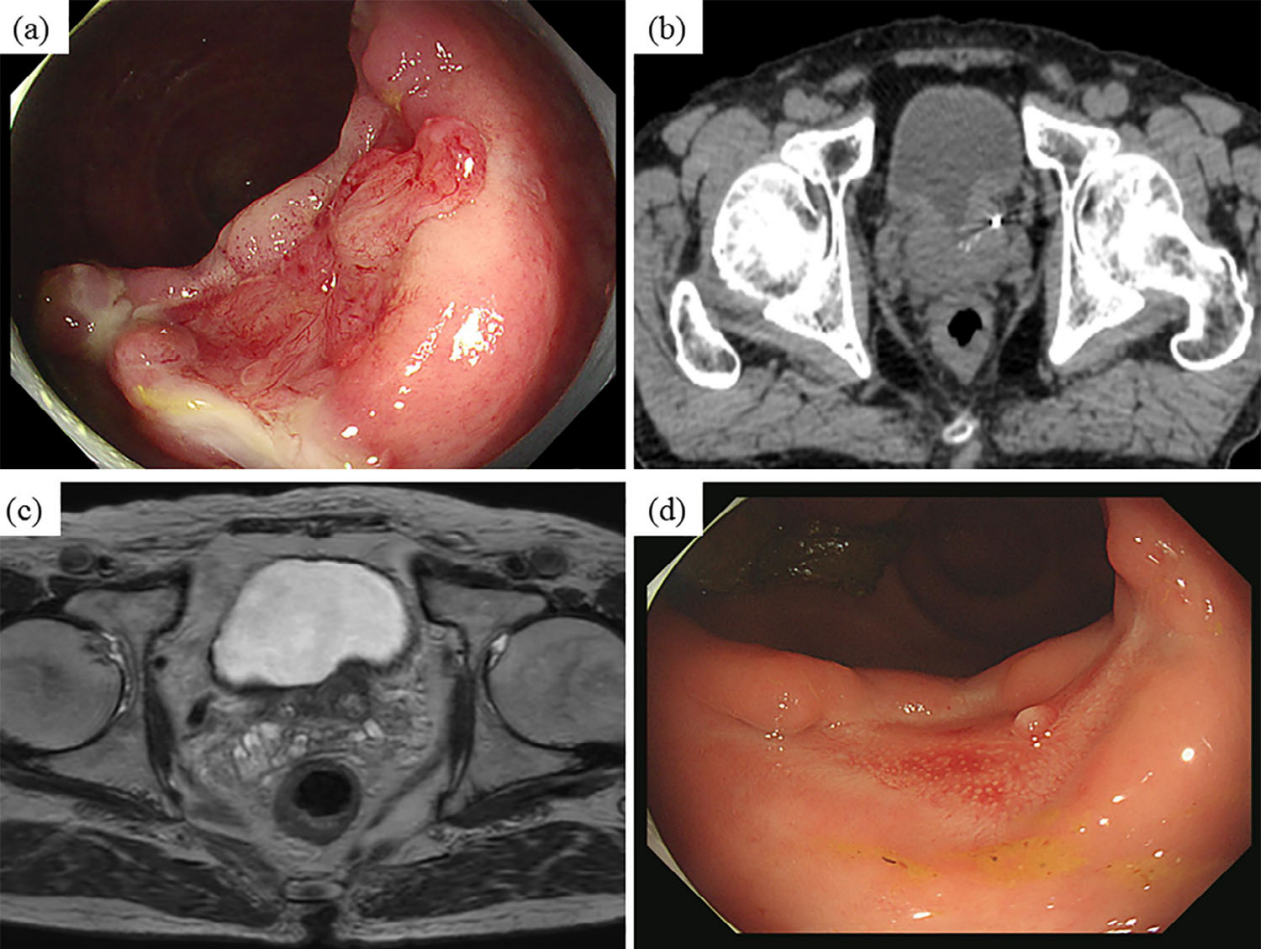
Colonoscopy (a), computed tomography (b), and magnetic resonance imaging (c) images obtained 2 months after the diagnosis indicating complete healing of the rectal ulcer. Scarring of the ulcer is observed during colonoscopy performed 6 months after hydrogel spacer insertion (d).

A 75‐year‐old man presented to the Department of Internal Medicine with hematochezia. He had been diagnosed with prostate cancer (stage 1) 1 month previously and had undergone gold marker injection and hydrogel spacer insertion 3 weeks previously to prepare for RT (Figure 1a). His medical history included allergic rhinitis, aortic regurgitation, and a sigmoid diverticulum. A physical examination revealed mild tenderness throughout the abdomen; however, obvious perineal irritation symptoms were not observed. Laboratory test results did not indicate any signs of anemia. Computed tomography (CT) identified a low‐density area that extended from the prostate to the rectal wall (Figure 1b). Axial T2‐weighted (Figure 1c) and sagittal T2‐weighted abdominal magnetic resonance imaging (MRI) revealed a hydrogel spacer between the anterior rectal wall and prostate. A colonoscopy revealed an approximately 2 cm ulcer in the rectum with bleeding around the hydrogel spacer (Figure 1d). The patient was diagnosed with a rectal ulcer with bleeding attributable to hydrogel spacer insertion. Because symptoms of peritoneal irritation and evidence of perforation of the gastrointestinal wall were not observed, magnesium oxide and sodium carbazochrome sulfonate were administered orally and a low‐residue diet was allowed (Figure 2a). The patient underwent conservative treatment as an outpatient. After 1 week of conservative treatment, the bloody stools gradually improved. One month after the diagnosis (2 months after insertion), the ulcer had improved. The center of the ulcer contained a jelly‐like substance similar to the hydrogel spacer (referred to as a jelly ulcer), and no signs of perforation were observed (Figure 2b,c). Two months after the diagnosis, the jelly ulcer was no longer visible and granulation formation was observed at the base of the ulcer during colonoscopy (Figure 3a). Complete healing of the rectal ulcer was confirmed by CT and MRI (Figure 3b,c). Therefore, RT for prostate cancer was initiated 4 months after insertion. During a colonoscopy performed 6 months after hydrogel spacer insertion, scarring of the ulcer was observed (Figure 3d). The patient has not experienced abdominal symptoms such as bloody stools since that time.

## DISCUSSION

RT is a standard treatment for prostate cancer. However, an overdose of radiation administered to the rectum can cause frequent defecation, defecation urgency, fecal incontinence, rectal bleeding, rectal ulcers, and fistula formation.^2^ Radiation proctitis is a complication of RT that can be difficult to treat; however, it can be avoided by hydrogel spacer insertion.^1^ After hydrogel spacer insertion, a space between the prostate and rectum is maintained for approximately 3 months; thereafter, the spacer is absorbed by the body. According to the Common Terminology Criteria for Adverse Events version 4, the incidence of grade 2 adverse events associated with hydrogel spacer insertion ranges from approximately 3.3% to 4.1%.^1^, ^3^ Four cases of rectal ulcers, including the present case, caused by hydrogel spacer insertion have been reported (Table 1).^4^, ^5^, ^6^ Only one patient was hospitalized; the other three patients were followed up as outpatients. In this case, the patient was allowed a low‐residue diet without antimicrobials and resumed eating a usual dish after the disappearance of hematochezia. Because the condition did not worsen, outpatient treatment was possible. All ulcers improved by 1–2 months after their diagnosis and did not recur.

**TABLE 1:** A case of rectal ulcer caused by hydrogel spacer insertion.

**Case**	**Reporter**	**Year**	**Age (years)**	**Sex**	**Stage**	**Gleason score **	**Insertion amount**	**Radiotherapy**	**Time from insertion to diagnosis**	**Time from diagnosis to mucosal improvement**	**History of sigmoid diverticula (Y/N)**	**CT**	**MRI**	**Colonoscopy**	**Hospitalization**	**Recurrence**	**Time from the insertion to resumption of radiotherapy**
1	Teh	2014	66	M	Unknown	3+4	Unknown	Brachytherapy	2 M	1 M	Y	Not listed	Not inspected	Ulcer at the anterior rectal no evidence of proctitis	Outpatient	None	Already treated
2	Iinuma	2019	63	M	Unknown	4+3	Unknown	Brachytherapy external radiation therapy	1 M	1 M	Y	Air in the space between the prostate and anterior rectal wall	Penetration of the rectum to the retroperitoneal space	Ulcer at the anterior rectal no evidence of proctitis	Outpatient	None	3 M later
3	Imai	2020	75	M	cT2aN0M0	3+3	Unknown	External radiation therapy	6 M	34 days	N	Air in the space between the prostate and anterior rectal wall	Infiltrated the anterior rectal wall	Ulcer with an exposed vessel at the anterior rectal no evidence of proctitis	Inpatient (5 days)	None	Already treated
4	This case	2024	75	M	cT1N0M0	4+4	10 mL	External radiation therapy	3 W	2 M	Y	Low density area in the space between the prostate and anterior rectal wall	High signal area within the rectal wall, between the prostate and the rectal wall	Ulcer at the anterior rectal no evidence of proctitis	Outpatient	None	4 M later

The mechanisms of rectal ulcers caused by hydrogel spacers are unknown; however, mechanical injury, ischemic injury, and radiation injury have been suggested.^4^ In previous cases, and in this case, infection and radiation proctitis were not observed. Spontaneous healing was observed without the administration of antimicrobial agents, except in the case reported by Teh et al.^4^, ^5^, ^6^ The source of infection was not reported by previous studies. Microcirculatory disturbance of the intestinal wall caused by thickening of the vascular lining and thrombus formation is the main pathogenesis of radiation proctitis.^7^ However, the effects of RT could not be associated with ulcers because ulcers have developed before and after RT in previous cases. Therefore, ischemic injury caused by excessive tension attributable to the hydrogel spacer or mechanical injury caused by perforation of the rectal wall by the injection needle may have caused tissue necrosis.

Hydrogel spacer insertion could predispose patients with sigmoid diverticula to rectal ulcers caused by ischemia and adhesions. Diverticula, which may be asymptomatic, are often associated with wall thickening, narrowing, and adhesions caused by chronic inflammation.^8^ Additionally, based on the multiple reports of sigmoidorectal fistulas^9^ and prostatic abscesses^10^ caused by inflammatory spillover from diverticulitis, the diverticulum could cause inflammation around the surrounding organs, including the rectum and the prostate. An adhesion could cause ulceration after hydrogel spacer insertion. In fact, three of the four reported patients had a history of sigmoid diverticula (Table 1). However, this is difficult to prove because of the small number of cases.

Because the number of patients with prostate cancer continues to increase, the number of cases similar to that of our patients is expected to increase in the future. Therefore, early detection is important. MRI or CT should be performed within 1 to 3 months after spacer insertion if the patient has a history of diverticulosis. Furthermore, an MRI or CT should be performed immediately if the patient has symptoms of abdominal pain or hematochezia.

In conclusion, we encountered a rare case of rectal ulceration caused by hydrogel spacer insertion in a patient with prostate cancer. Although hydrogel spacer insertion prevents radiation proctitis, it should be recognized as the potential cause of adverse events.

## CONFLICT OF INTEREST STATEMENT

None.

## ETHICS STATEMENT

N/A.

## PATIENT CONSENT STATEMENT

The patient has provided written informed consent for the publication of this report.

## REGISTRY AND THE REGISTRATION NO. OF THE STUDY/TRIAL

N/A.

## Data Availability

All data generated or analyzed during this study are included in this published article.
